# PHNQ from *Evechinus chloroticus* Sea Urchin Supplemented with Calcium Promotes Mineralization in Saos-2 Human Bone Cell Line

**DOI:** 10.3390/md18070373

**Published:** 2020-07-19

**Authors:** Yakun Hou, Alan Carne, Michelle McConnell, Sonya Mros, Elena A. Vasileva, Natalia P. Mishchenko, Keegan Burrow, Ke Wang, Adnan A. Bekhit, Alaa El-Din A. Bekhit

**Affiliations:** 1 Department of Food Science, University of Otago, Dunedin 9016, New Zealand; yakun.hou@postgrad.otago.ac.nz (Y.H.); keeganburrow@gmail.com (K.B.); wanke683@student.otago.ac.nz (K.W.); 2 Department of Biochemistry, University of Otago, Dunedin 9016, New Zealand; alan.carne@otago.ac.nz; 3 Department of Microbiology and Immunology, University of Otago, Dunedin 9016, New Zealand; michelle.mcconnell@otago.ac.nz (M.M.); Sonya.Mros@otago.ac.nz (S.M.); 4 G.B. Elyakov Pacific Institute of Bioorganic Chemistry, Far Eastern Branch of Russian Academy of Sciences, 690041 Vladivostok, Russia; vasilieva_el_an@mail.ru (E.A.V.); mischenkonp@mail.ru (N.P.M.); 5 Department of Pharmaceutical Chemistry, Faculty of Pharmacy, Alexandria University, Alexandria 21521, Egypt; adnbekhit@pharmacy.alexu.edu.eg; 6 Pharmacy Program, Allied Health Department, College of Health Sciences, University of Bahrain, Sakheer P.O. Box 32 038, Bahrain

**Keywords:** PHNQ, sea urchin, spinochrome, cytotoxicity, Saos-2 cells, mineralized nodule formation

## Abstract

Polyhydroxylated naphthoquinones (PHNQs), known as spinochromes that can be extracted from sea urchins, are bioactive compounds reported to have medicinal properties and antioxidant activity. The MTT (3-(4,5-Dimethylthiazol-2-yl)-2,5-diphenyltetrazolium bromide) cell viability assay showed that pure echinochrome A exhibited a cytotoxic effect on Saos-2 cells in a dose-dependent manner within the test concentration range (15.625–65.5 µg/mL). The PHNQ extract from New Zealand sea urchin *Evechinus chloroticus* did not induce any cytotoxicity within the same concentration range after 21 days of incubation. Adding calcium chloride (CaCl_2_) with echinochrome A increased the number of viable cells, but when CaCl_2_ was added with the PHNQs, cell viability decreased. The effect of PHNQs extracted on mineralized nodule formation in Saos-2 cells was investigated using xylenol orange and von Kossa staining methods. Echinochrome A decreased the mineralized nodule formation significantly (*p* < 0.05), while nodule formation was not affected in the PHNQ treatment group. A significant (*p* < 0.05) increase in mineralization was observed in the presence of PHNQs (62.5 µg/mL) supplemented with 1.5 mM CaCl_2_. In conclusion, the results indicate that PHNQs have the potential to improve the formation of bone mineral phase in vitro, and future research in an animal model is warranted.

## 1. Introduction

Sea urchin shell contains bioactive polyhydroxylated naphthoquinone (PHNQ) pigments and derivatives of PHNQ substituted with ethyl, acetyl, methoxy or amino groups, that are known as echinochrome and collectively as spinochromes [1,2,3]. A wide range of biological activities has been ascribed to the spinochromes, including antioxidant, anti-microbial, and anti-inflammatory activity. A number of studies have reported that PHNQs are strong antioxidants that can block a number of free radical reactions, inhibit lipid peroxidation, and chelate metal ions [1,4,5,6]. In addition, PHNQs have exhibited antibacterial activity against both model human pathogenic bacteria and marine bacterial strains [7,8]. PHNQs have also demonstrated protective effects against several human health disorders such as cardiovascular diseases and eye conditions, as well as displaying anti-inflammatory activity [7,9]. Among all the known PHNQs, echinochrome A is the most well studied compound in terms of its bioactivities. Echinochrome A is the active substance in the Russian drug Histochrome [10], and has been found to exhibit additional biological effects other than antioxidant activity, including anti-fibrosis, anti-diabetic, anti-allergic, anti-acetylcholinesterase, mitochondria-protective and gastro-protective effects [11,12,13,14,15].

The formation and remodelling of bone are essential for the development, maturation, maintenance, and repair of bones. The formation of bone involves a complex series of events including the proliferation and differentiation of osteoblast cells, eventually leading to the formation of a mineralized nodule [16]. Osteoblast cells play a significant role in bone formation. Based on studies of in vitro bone nodule formation, the process starts with proliferation of osteoblast cells, followed by their differentiation and matrix mineralization to form new bone matrix [17]. The breakdown of the bone growth and remodelling pathways can increase the risk of bone disorders such as osteoporosis, which is considered to be the most common bone disorder and remains an increasing health problem [18]. At the cellular level, osteoporosis is caused by an imbalance between bone formation involving osteoblasts, and bone resorption involving osteoclasts [19]. An ideal strategy to treat osteoporosis is to inhibit bone resorption by osteoclasts and increase bone formation by osteoblasts [19].

Some treatments such as calcium (Ca) and vitamin D supplements, or hormone therapies, have been found to not completely stop the progression of osteoporosis [20]. Recently, there has been growing interest in the treatment of osteoporosis with natural products derived from traditional Chinese medicine [19,21]. Echinacoside, isolated from *Cistanche tubulosa* (Schrenk) R. Wight (Orobanchaceae parasitic plant, common name Roucongrong) stems, has been reported to cause a substantial increase in cell proliferation, alkaline phosphatase (ALP) activity, secretion of collagen I, osteocalcin levels, and to enhance mineralization in osteoblasts in vitro using MC3T3-E1 cells, at a concentration range from 0.01 to 10 nmol/L (*p* < 0.05) [22]. Vanillic acid, a phenolic acid isolated from *Sambucus williamsii* Hance (*Caprifoliaceae* family commonly known as elderberry), has been used for the treatment of bone and joint disease in China for thousands of years [23]. A number of natural products from a variety of fruits and vegetables, such as rutin and quercetin that have also been evaluated for their potential in management of osteo-degenerative disorders, were reported to increase ALP activity by about 150% and 240% and enhance mineralization by up to 110% and 200%, respectively, compared to control, in isolated mouse bone-marrow-derived mesenchymal stem cells in vitro [24].

Extracts from sea urchin shell and spine have been reported to have medicinal properties [25,26]. Chinese pharmacopoeia, the main reference for traditional Chinese medicinal, recorded that sea urchin dry calcareous shells have the function of acting as a decongestant (“Ruan jian san jie”, ‘resolving phlegm, elimination swelling, expectorate sputum accumulation’) [25]. The edible roe of *E. chloroticus*, a sea urchin species that is endemic to New Zealand, has been considered a local delicacy and has potential for export to other countries, including Japan and China [3,8]. Commercial harvesting of *E. chloroticus* roe generates a considerable amount of shell and spine as waste, that can contribute to environmental issues [3]. Thus, the potential health promotion effect of PHNQ extracts from *E. chloroticus* may add value to the shell and spine waste and potentially reduce environmental issues.

Even though many studies have investigated the bioactivities of PHNQs, to the best of our knowledge, the effect of PHNQ on osteoblast cells and the formation of mineralized nodules has not been reported previously. In vitro cytotoxicity assays measure whether a test compound is toxic to cells in culture by determining the number of viable cells remaining after an incubation period. The general aim of the present study was to investigate whether PHNQs from New Zealand sea urchin *E*. *chloroticus* have any effect on bone tissue mineralization in human osteogenic sarcoma cells (Saos-2 cells) and whether PHNQ supplemented with CaCl_2_ promotes bone tissue mineralization in the Saos-2 human bone cell line.

## 2. Results

### 2.1. Extraction of PHNQs from E. choloticus Spine

**Figure 1:**
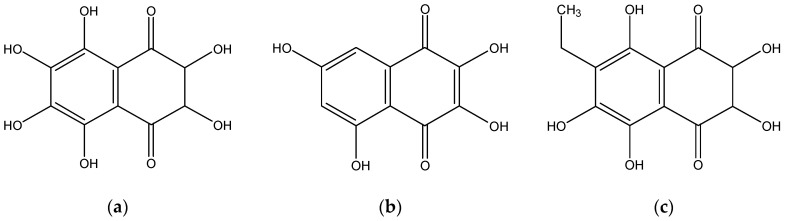
Structure of major polyhydroxylated naphthoquinones (PHNQs) in *E. chloroticus* spine. (**a**) spinochrome E; (**b**) spinochrome B; (**c**) echinochrome A.

The PHNQs in *E. chloroticus* spine extracted by ethyl acetate were characterised using high-performance liquid chromatography (HPLC) with a diode-array detector (DAD) and mass spectrometry (MS). Three major PHNQs including spinochrome E, spinochrome B and echinochrome A (each representing more than 5% of the total PHNQ content), and five minor PHNQs including spinamine E, spinochrome C, spinochrome A, echinamine A and echinamine B were identified by direct comparison of their ESI-MS and absorption spectra with authentic samples isolated from *Mesocentrotus nudus* [3]. The identification of these PHNQs was based on their retention time and UV/Vis absorption data, compared to those of published data. A typical chromatogram can be found in our previous study [3]. The structures of major PHNQs in *E. chloroticus* spine are shown in Figure 1.

### 2.2. Cytotoxic Activity of E. chloroticus PHNQ Extract

**Figure 2:**
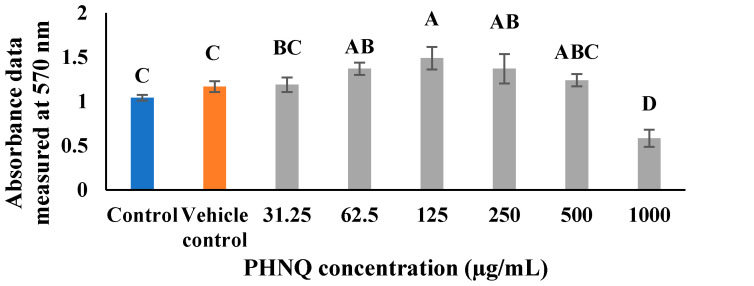
Absorbance data measured at 570 nm of Saos-2 cells treated with different concentrations of PHNQ extract as determined using the MTT (3-(4,5-Dimethylthiazol-2-yl)-2,5-diphenyltetrazolium bromide) assay as described in Section 4.4 after 48 h incubation. Data represent the mean ± standard deviation of three independent experiments, each concentration tested in duplicates. Means with different letters for the group with different concentrations are significantly different (*p* < 0.05). DMSO (3.725% *v*/*v*) was used as vehicle control. Sodium dodecyl sulfate (SDS) at 10% (*w*/*v*) in final media was used as a positive control, for which no viable cells were observed after 48 h incubation.

As shown in Figure 2, the viable cell percentage for 1000 µg/mL PHNQ was significantly lower than that of the control groups after incubating the cells for 48 h with or without PHNQ extract (*p* < 0.05), as described in Section 4.4. PHNQ extract at this concentration reduced the mean viable cell percentage to 57.16% of the control, which was considered as being toxic to Saos-2 cells. The PHNQ extract at a final concentration up to 500 µg/mL was considered to be not toxic towards Saos-2 cells after 48 h incubation, indicating that up to this concentration could be used for the evaluation of the effect of PHNQs on mineralization in Saos-2 cells.

The half maximal effective concentration (EC_50_) of the PHNQ extract was 1018.6 ± 51.4 µg/mL. At PHNQ extract concentrations between 62.5 to 250 µg/mL, the number of viable cells was significantly higher than the number of control cells (*p* < 0.05) after 48 h incubation. However, preliminary experiments showed that after 15 days incubation, very few viable cells were observed microscopically in the 125 and 250 µg/mL treatment groups while more cells were observed in the control group, showing that, at these concentrations, PHNQ may still be toxic to Saos-2 cells over long-term incubation. Therefore, 62.5 µg/mL was chosen as the highest treatment concentration of PHNQ extract, along with 31.25 and 15.625 µg/mL PHNQ extract for the following experiments.

### 2.3. The Effect of CaCl_2_, E. choloroticus PHNQ Extract and Echinochrome A on the Proliferation of Saos-2 Cells

**Figure 3:**
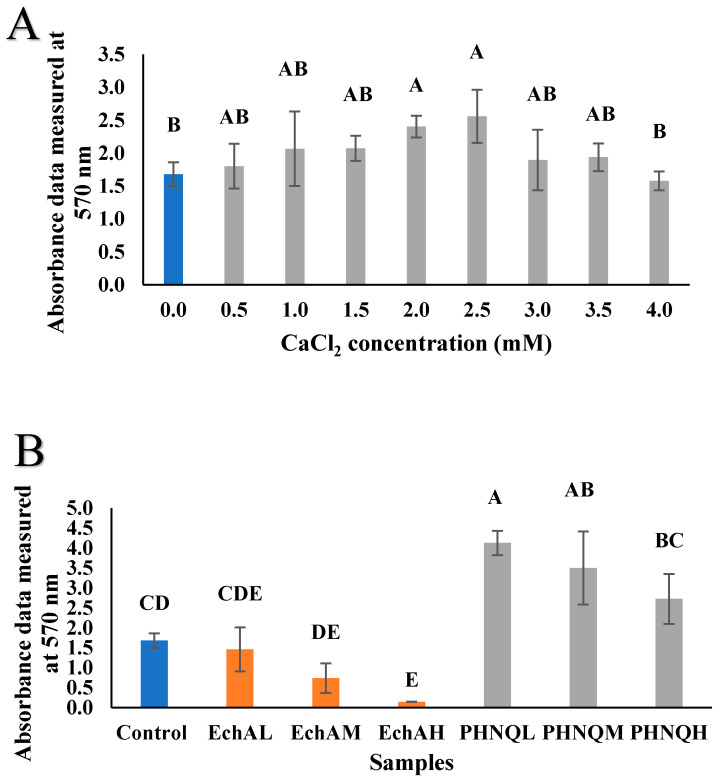
Absorbance data measured at 570 nm of Saos-2 cells treated with different concentrations of CaCl_2_ (**A**), echinochrome A and PHNQ (**B**), as determined using the MTT assay as described in Section 4.5 after 21 days of incubation. Data represent the mean ± standard deviation of three independent experiments; each concentration was tested in duplicates. Means with different letters are significantly different (*p* < 0.05), determined by one-way ANOVA. Sodium dodecyl sulfate (SDS) at 10% (*w*/*v*) in final media was used as a positive control (no viable cells were evident). EchA refers to echinochrome A, with H: 62.5 µg/mL; M: 31.25 µg/mL; L: 15.625 µg/mL. PHNQH, PHNQM and PHNQL refer to PHNQ at concentrations of 62.5, 31.25 and 15.625 µg/mL, respectively.

As reported in Figure 3A, a range of 0–4.0 mM CaCl_2_ was applied to the Saos-2 cells, as described in Section 4.5. CaCl_2_ showed no cytotoxic effect on Saos-2 cells after 21 days at increasing doses and most groups were comparable to the cells only control. Supplementation with CaCl_2_ at 2.0 mM and 2.5 mM exhibited an increase in the number of viable Saos-2 cells after 21 days incubation (about a 1.5-fold increase compared to the control group, *p* < 0.05). Echinochrome A exhibited a cytotoxic effect on Saos-2 cells in a dose-dependent manner within the range of concentrations tested (Figure 3B), and a higher concentration resulted in proportionately fewer viable cells remaining. Only 8.61% of the cells remained viable after 21 days incubation with the highest concentration (62.5 µg/mL) of echinochrome A, and about 44.1% remained viable after treatment with 31.25 µg/mL of echinochrome A. With the lowest concentration of echinochrome A, 86.8% of the cells remained viable and hence could be considered not toxic to Saos-2 cells. The EC_50_ for echinochrome A was determined to be 32.01 ± 8.02 µg/mL. In the present study, the PHNQ extract did not show any cytotoxicity after 21 days of incubation at all test concentrations (62.5, 31.25 and 15.625 µg/mL). Therefore, all subsequent experiments were performed with PHNQ extract at these concentrations. PHNQ extract showed a proliferation effect in a dose-dependent manner, as the lowest concentration of PHNQ extract exhibited a better proliferation effect, and the highest concentration of PHNQ extract increased the viable cell number 1.5-fold, while the lowest concentration of PHNQ extract increased the viable cell number 2.5-fold compared to the cells-only control. The lowest and medium PHNQ extract concentrations increased cell growth significantly compared to the control (*p* < 0.05).

### 2.4. Effect of Adding CaCl_2_ with Echinochrome A and E. choloroticus PHNQ Extract on Cell Viability

**Figure 4:**
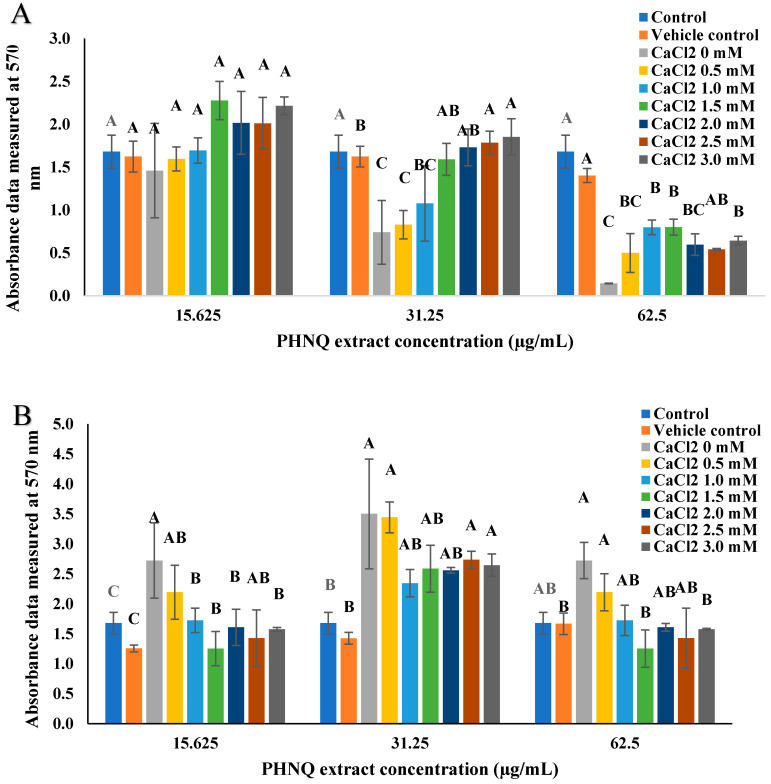
Absorbance data measured at 570 nm of Saos-2 cells treated with echinochrome A (**A**) and PHNQ extract (**B**) with different concentrations of CaCl_2_, as determined using the MTT assay as described in Section 4.4 after 21 days incubation. Data represent the mean ± standard deviation of three independent experiments; each concentration was tested in duplicates. Columns with different letters are significantly different (*p* < 0.05) for different concentrations of echinochrome A or PHNQ, determined by one-way ANOVA. Sodium dodecyl sulfate (SDS) at 10% (*w*/*v*) in final media was used as a positive control, with no viable cells evident. Cytotoxicity of PHNQ on Saos-2 cells was measured by MTT assay. Same volume of DMSO was used as treatment group for the treatment in vehicle control group.

As shown in Figure 4, adding CaCl_2_ increased the number of viable cells at the high and medium concentrations, but did not show any effect at the low concentration of echinochrome A. For the high concentration of echinochrome A, adding 1.0 and 1.5 mM CaCl_2_ resulted in less cytotoxicity compared to other concentrations tested, but the viable cell percentage was under 50%, which was considered to be toxic and unacceptable. For the medium concentration of echinochrome A, adding CaCl_2_ increased the viable cell percentage from about 50% to over 80% when the CaCl_2_ concentration was above 1.0 mM. The number of viable cells decreased after adding CaCl_2_ with PHNQ at all three PHNQ concentrations (Figure 4B), but treatment with PHNQ and CaCl_2_ did not cause any cytotoxicity to Saos-2 cells. In Figure 4, the concentrations of 15.625, 31.25, and 62.5 µg/mL of PHNQ extract showed viable cell percentages of approximately 250, 170, and 210%, and were similar to those obtained in Figure 3. These similarities could highlight the repeatability of the assay. The repeatability of the data in Section 2.4 was analysed using Crossed Gage R&R Study (crossed) using Minitab 17.0 software (Minitab Pty Ltd., Sydney, Australia). The Total Gage R&R for PHNQ extract and echinochrome A equals 29% and 27.61%, respectively of the study variation. The Total Gage R&R %Contribution is acceptable in biological test ranging from 0–30%.

### 2.5. Effect of CaCl2 on Minerlization of Saos-2 Cells and the Mineralization Assay Evaluation

**Figure 5:**
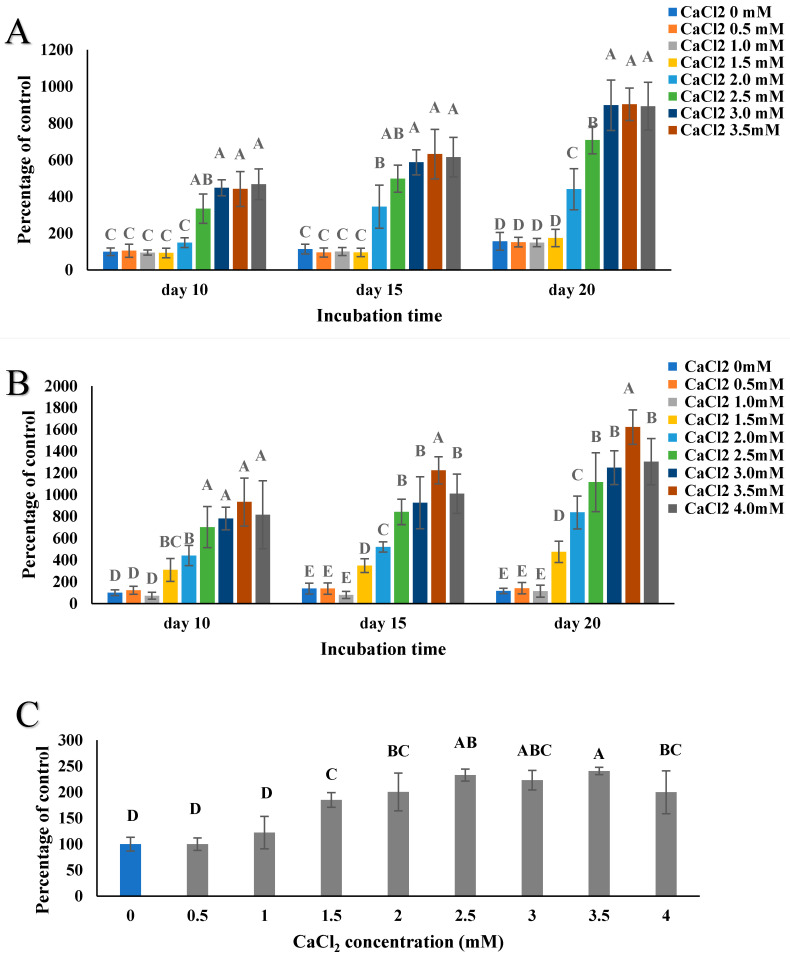
Formation of mineralized nodules by Saos-2 cells treated with different concentrations of CaCl_2_. (**A**) measured as a percentage of control (with excitation and emission wavelengths of 440 and 610 nm, respectively), after staining with xylenol orange (at 20 μmol for 24 h) at different timepoints. Percentage of control was calculated according to the quotation in Section 4.5. (**B**) Measured as a percentage of control after staining with xylenol orange (at 20 μmol for 24 h) at different timepoints (Quantitative image analysis by ImageJ, see details in Appendix A). Percentage of control was calculated according to the quotation in Appendix A. (**C**) Measured as a percentage of control after staining with von Kossa (Quantitative image analysis by ImageJ). Percentage of control was calculated according to the quotation in Appedix A. Data represent the mean ± standard deviation of three independent experiments, each concentration was tested in duplicate. Means with different letters are significantly different (*p* < 0.05), determined by one-way ANOVA using Tukey’s honesty test at *p*-value < 0.05. Cells only (CaCl_2_ = 0 mM) were used as a control.

**Figure 6:**
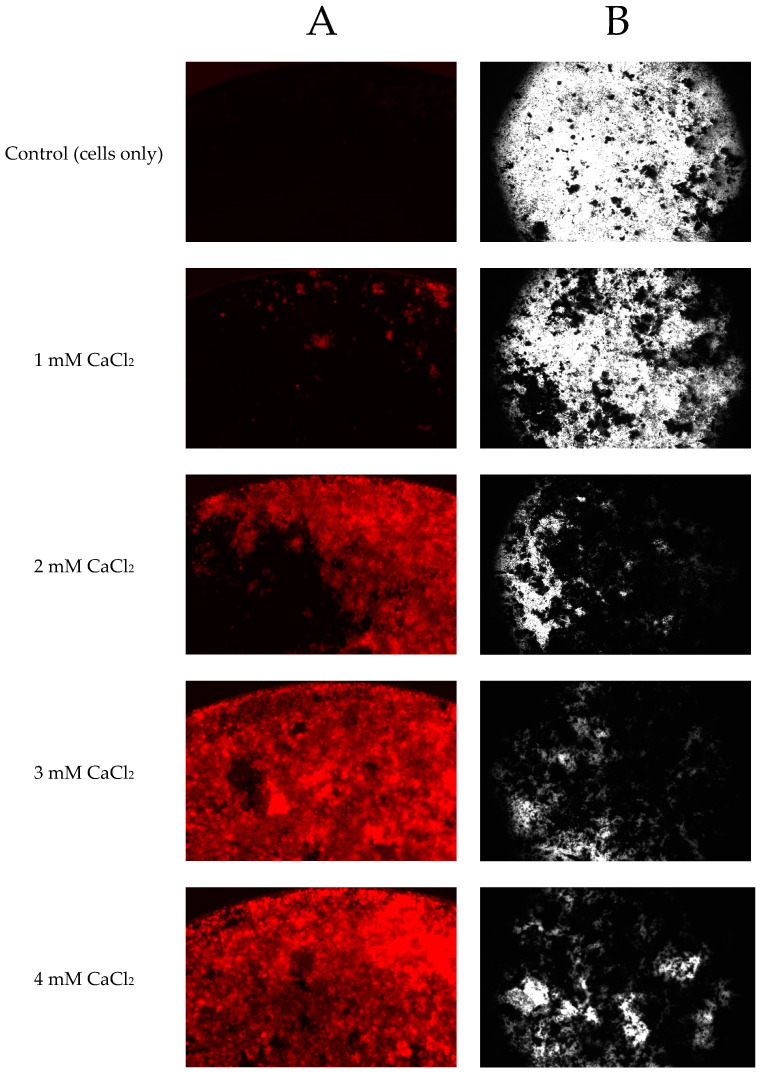
Representative examples of stained images of the formation of mineralized nodules by Saos-2 cells treated with CaCl_2_ at 1, 2, 3, 4 mM. Cells stained with either (**A**) xylenol orange (at 20 μmol for 24 h) at day 20 or using the (**B**) von Kossa method (silver nitrate) at day 21. Images were taken with a Nikon DS-Qi2 Camera fitted to a Nikon (ECLIPSE, Ti2) inverted fluorescent (or light) microscope. An exposure time of 166 ms was used for xylenol orange.

The results showed that the staining intensity was increased as the CaCl_2_ concentration increased for both the fluorescent staining method and the von Kossa staining method (Figure 5A,B). The control group (cells only) had very weak fluorescent staining, which was comparable to the von Kossa staining (Figure 6). The correlation between plate reader data (Figure 5A) and image analysis data (Figure 5B) for fluorescent staining was high (r = 0.934, *p* < 0.001). The fluorescent staining method (Figure 5B) and the von Kossa staining method (Figure 5C) had the same trend (r = 0.971, *p* < 0.001) (correlations among data obtained were calculated using Pearson’s correlation coefficient *r*).

The effect of time and CaCl_2_ concentration on the formation of mineralized nodules is presented in Figure 5A. Treatment of the cells with CaCl_2_ increased the mineralization in a time-dependent manner and there was a significant difference in the formation of mineralized nodules between different time points (*p* < 0.05). CaCl_2_ showed the highest mineralization effect on day 21 and lowest effect on day 10. Treatment with CaCl_2_ increased the mineralized nodules in a dose-dependent manner at concentrations of 1.5–3.0 mM (Figure 5A) and at concentrations of 1.5–2.5 mM (Figure 5B). With the CaCl_2_ concentration at 3.5 mM and 4.0 mM, no more mineralized nodules were observed to have formed compared to CaCl_2_ at 3 mM, suggesting a saturation effect.

### 2.6. Effect of PHNQ Extract and Echinochrome A on Mineralization of Saos-2 Cells

**Figure 7:**
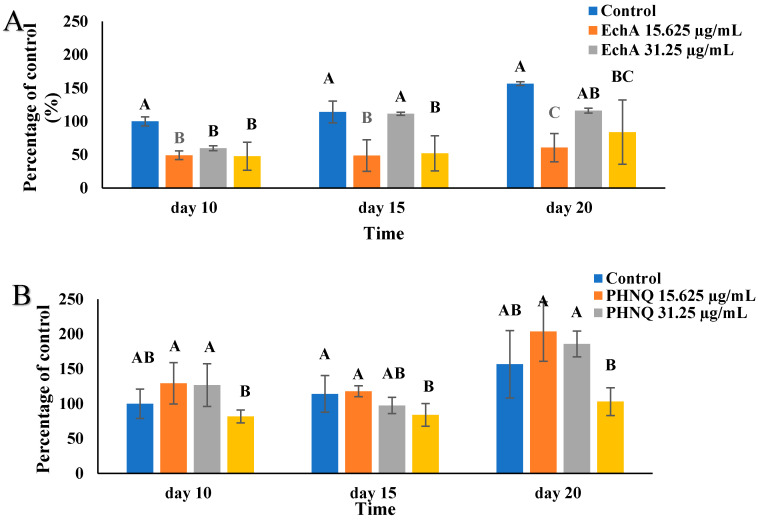
Formation of mineralized nodules by Saos-2 cells treated with different concentrations of (**A**) Echinochrome A and (**B**) PHNQ extract. Measured as a percentage of control (with excitation and emission wavelengths of 440 and 610 nm, respectively), after staining with xylenol orange (at 20 μmol for 24 h). Data represent the mean ± standard deviation of three independent experiments, each concentration was tested in duplicate. Means with different letters on the same timepoint are significantly different (*p* < 0.05), as determined one-way ANOVA using Tukey’s honesty test at *p-*value < 0.05. Cells-only were used as a control (EchA: echinochrome A). Percentage of control was calculated according to the quotation in Section 4.6.

The mineralized nodule formation in Saos-2 cells cultured in treatment medium with echinochrome A or PHNQ extract at different timepoints is shown in Figure 7. On day 15 and day 20, the presence of the medium concentration (31.25 µg/mL) of echinochrome A showed a comparable mineralization effect to that of the control (cells only) group, while the lowest and highest concentrations of echinochrome achieved a significantly lower mineralization than the cells-only control (*p* < 0.05) (Figure 7).

**Figure 8:**
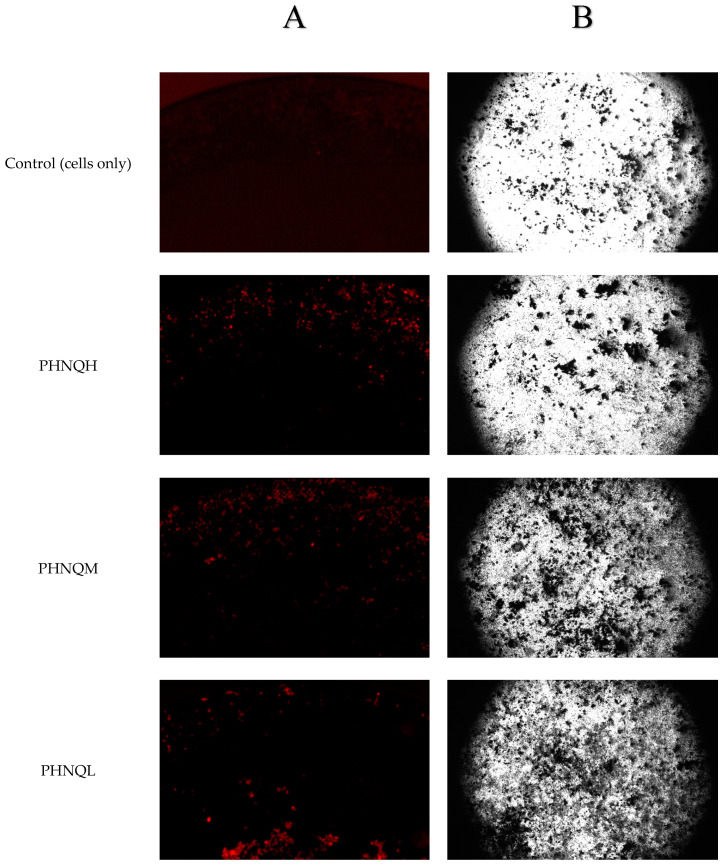
Representative stained images of the formation of mineralized nodules by Saos-2 cells treated with different concentrations of PHNQ extract and cells-only control. Stained with either (**A**) xylenol orange (at 20 μmol for 24 h) at day 20 or using (**B**) the von Kossa method (silver nitrate) at day 21. Images were taken with a Nikon DS-Qi2 Camera fitted to a Nikon (ECLIPSE, Ti2) inverted fluorescent (or light) microscope. An exposure time of 166 ms was used for xylenol orange. (PHNQH: 62.5 µg/mL PHNQ; PHNQM: 31.25 µg/mL PHNQ; PHNQL: 15.625 µg/mL PHNQ).

Treatment with PHNQ extract did not change the mineralization nodule formation on day 20 (*p* < 0.05) for all PHNQ concentrations tested. The low and medium concentration groups had a tendency to increase the mineralization, and the high concentration group had a tendency to decrease mineralized nodule formation in Saos-2 cells. It is worth mentioning that based on the proliferation experiment, the numbers of Saos-2 cells were increased when treated with low or medium concentrations of PHNQ extract. In addition, the lowest mineralized nodule formation effect was observed with the highest concentration of PHNQ extract at all three time points, but it showed a significant decrease only on day 15 (*p* < 0.05) (Figure 7B). Stained images of the formation of mineralized nodules by Saos-2 cells treated with the highest, medium, and lowest concentrations of PHNQ extract and control (cells-only) are shown in Figure 8. The images showed that there were more mineralized nodules stained by von Kossa for the medium and the lowest PHNQ concentration treatment groups than with the highest PHNQ concentration group.

### 2.7. Effect of PHNQ on Mineralization in the Presence of CaCl_2_

**Figure 9:**
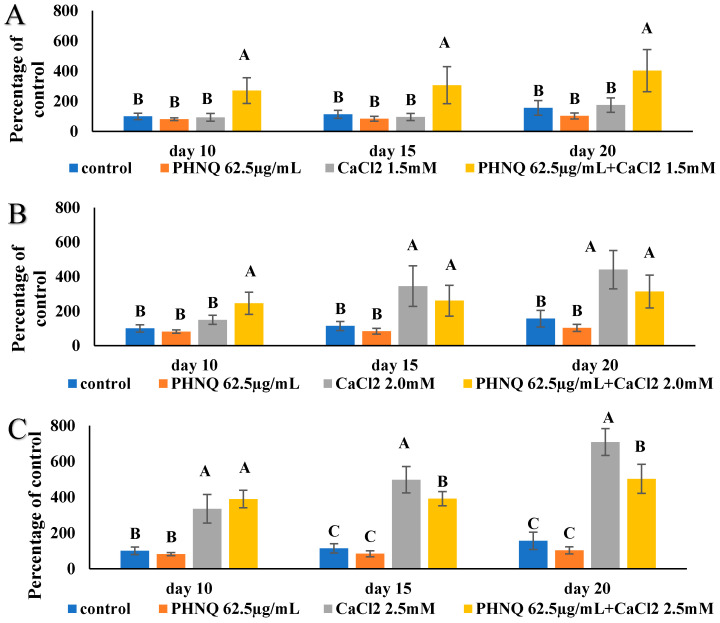
Formation of mineralized nodules by Saos-2 cells treated with PHNQH and different concentrations of CaCl_2__._ (**A**) PHNQH + 1.5 mM CaCl_2_ (**B**) PHNQH + 2.0 mM CaCl_2_ (**C**) PHNQH + 2.5 mM CaCl_2_ (compared with PHNQ extract alone and CaCl_2_ alone. Measured as a percentage of the control (with excitation and emission wavelengths of 440 and 610 nm, respectively), after staining with xylenol orange (at 20 μmol for 24 h). Data represent the mean ± standard deviation of three independent experiments, each concentration was tested in duplicate. Means with different letters are significantly different (*p* < 0.05), as determined by one-way ANOVA using Tukey’s honesty test at *p*-value < 0.05.

**Figure 10:**
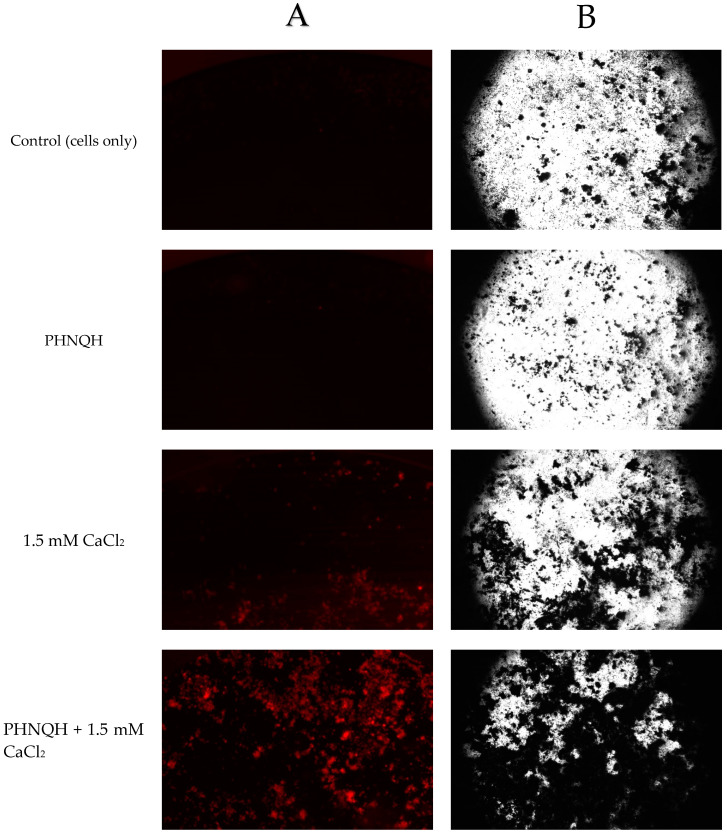
Representative stained images of the formation of mineralized nodules by Saos-2 cells treated with PHNQH, CaCl_2_ and PHNQH + CaCl_2_. Stained with either (**A**) xylenol orange (at 20 μmol for 24 h) at day 20 or using (**B**) the von Kossa method (silver nitrate) at day 21. Images were taken with a Nikon DS-Qi2 Camera fitted to a Nikon (ECLIPSE, Ti2) inverted fluorescent (or light) microscope. An exposure time of 166 ms was used for xylenol orange. (PHNQH: 62.5 µg/mL PHNQ).

**Figure 11:**
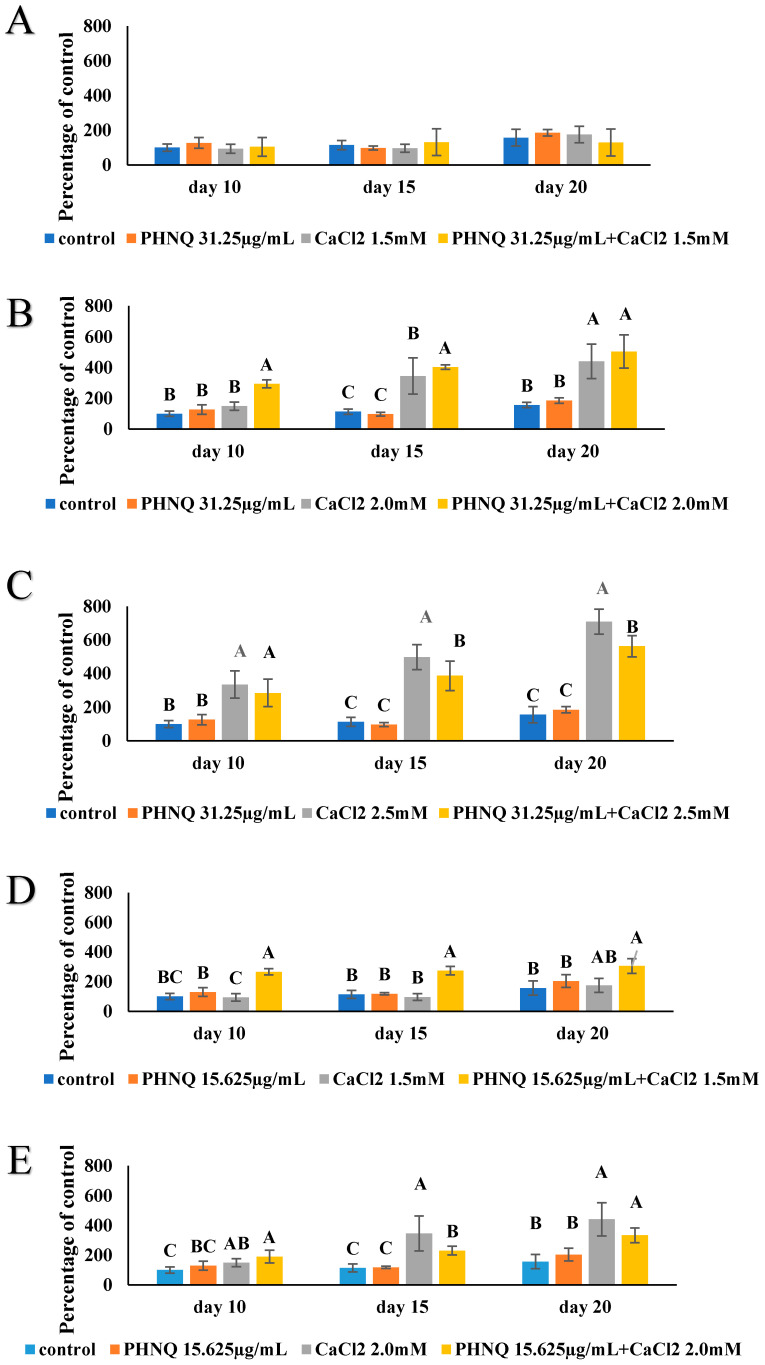

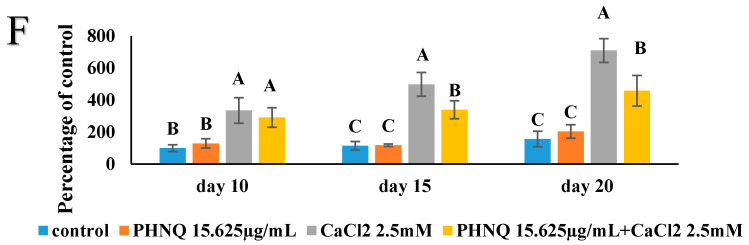
Formation of mineralized nodules by Saos-2 cells treated with PHNQM and PHNQL with added different concentrations of CaCl_2_. (**A**) PHNQM + 1.5 mM CaCl_2_ (**B**) PHNQM + 2.0 mM CaCl_2_ (**C**) PHNQM + 2.5 mM CaCl_2_ (**D**) PHNQL + 1.5 mM CaCl_2_ (**E**) PHNQL + 2.0 mM CaCl_2_ (**F**) PHNQL + 2.5 mM CaCl_2_ compared with PHNQ extract alone and CaCl_2_ alone. Measured as percentage of control (with excitation and emission wavelengths of 440 and 610 nm, respectively), after staining with xylenol orange (at 20 μmol for 24 h). Data represent the mean ± standard deviation of three independent experiments, each concentration was tested in duplicate. Means with different letters are significantly different (*p* < 0.05), as determined by one-way ANOVA using Tukey’s honesty test at p-value < 0.05. Cells only (CaCl_2_ = 0 mM) at day 10 used as a control.

In the absence of CaCl_2_, the high concentration of PHNQ did not result in any increase in mineralized nodule formation at all three timepoints (day 10, day 15 and day 20, Figure 7B), even though at this concentration, the PHNQ extract did not show any cytotoxicity to Saos-2 cells. When 1.5 mM CaCl_2_ was added to this level of PHNQ extract in the media, a significant increase in mineralization from 81.73 ± 9.13% (relative fluorescence units) to 270.74 ± 84.88% on day 10, 83.83 ± 16.28% to 306.82 ± 122.61% on day 15 and 103.01 ± 20.04% to 403.20 ± 140.14% on day 20 was obtained (Figure 9A). Such a two- to three-fold amplification of mineralization caused by adding a supplement of 1.5 mM CaCl_2_ was confirmed by both an increase in fluorescence staining and von Kossa staining on day 20 (Figure 10). Supplementation with 2.0 and 2.5 mM CaCl_2_ also resulted in amplification of mineralization compared with the PHNQ-only group, but it should be noted that 2.0 and 2.5 mM CaCl_2_ alone also had a significant stimulation effect on the mineralized nodule formation compared to control. Thus, there is no augmentation effect of CaCl_2_ at the concentrations of 2.0 and 2.5 mM in the presence of PHNQs. When 2.0 mM CaCl_2_ was added and co-incubated with a medium concentration of PHNQ extract, a significant amplification (*p* < 0.05) of mineralization on day 15 (167.74%) and a non-significant increase on day 20, as reflected by an increase in fluorescent staining, as measured by plate reader, was observed. For the low concentration of PHNQ, adding 1.5 mM CaCl_2_ caused a significant increase (*p* < 0.05) in the mineralized nodule formation on day 10 and day 15, which was 136.96% and 156.22%, respectively (Figure 11). Supplementation with 2.0 and 2.5 mM CaCl_2_ also enhanced mineralization, but the effect on the low concentration of PHNQ with adding 2.0/2.5 mM CaCl_2_ was lower than that of CaCl_2_ only (*p* < 0.05) (Figure 11E,F).

## 3. Discussion

In the present study, viable cells were determined using the MTT assay that relies on the ability of mitochondrial dehydrogenases to oxidize a thiazolyl blue tetrazolium bromide compound (MTT; 3-[4,5-dimethylthiazol-2-yl]-2,5-diphenyltetrazolium bromide) to an insoluble blue formazan product [27,28]. Considering that the mineralized nodule formation assay takes up to 21 days, a further MTT assay was carried out at 21 days of incubation followed by the first 48-h testing to determine the effect of *E. chloroticus* PHNQ extract, echinochrome A and CaCl_2_ on the viability of Saos-2 cells before conducting the mineralization assays.

The determination of the cytotoxicity of PHNQs from other species of sea urchins has been reported previously in the literature [7,29,30]. Because PHNQs have been interesting candidates for potential effects on human health, one previous study investigated the cytotoxicity of PHNQs on human HeLa cells using the MTT assay [7] to evaluate the possibility of future pharmacological application. To evaluate the possibility of future pharmacological application, PHNQ extracted from the Indian Ocean sea urchin *Echinometra mathaei* (Blainville, 1825) was tested [7]. The results showed a slight decrease in cell viability at high concentrations and only spinochrome E was classified as a moderate cytotoxic compound EC_50_ < 90 μg/mL), while other PHNQs such as spinochrome B, spinochrome A and echinochrome A were found to exhibit less toxicity (EC_50_ < 120 μg/mL) [7], that is at least 10-fold different compared to the present study. However, it is worth noting that the human HeLa cells were exposed to these isolated PHNQs for 24 h but in the present study, the exposure time was either 48 h or 21 days. In the present study, the 48-h MTT assay showed that the cytotoxicity of the PHNQ extract (EC_50_ > 1000 μg/mL) was less than the separated PHNQ in the study of Brasseur et al., [7] but it should be noted that different cell lines may react differently to the same compound. The 21-day MTT assay exhibited that echinochrome A (EC_50_ = 32.01 ± 8.02 µg/mL), as an individual PHNQ, was more toxic to Saos-2 cells compared to PHNQ extract (EC_50_ > 62.5 μg/mL), indicating twice the toxicity. Sung et al. [30] found that commercial histochrome (echinochrome A) did not exhibit significant (*p* < 0.05) toxicity on A7r5 cells (rat aortic vascular smooth muscle cell line) and H9c2 cells (rat cardiomyoblasts) up to 100 μM (26.6 µg/mL) for 24 h. Vasileva and Mishchenko [27] investigated the toxicity of various PHNQs (echinochrome A, echinamines A and B, and spinochromes A and B) against sea urchin egg cells and obtained inhibitory concentration (IC) values ranging between 10 and 100 µg/mL, depending on the stage of development of the cells. From this study, the cytotoxic activity of the PHNQs was ranked as echinochrome A > echinamines A and B > spinochromes A and B. In the present study, the concentration of PHNQ extract that was toxic to Saos-2 cells was found to be higher than that of echinochrome A (at least two times higher), indicating that spinochromes are less cytotoxic than echinochrome A. Alternatively, the results imply that other compounds (spinochromes or non-PHNQ compounds) in the PHNQ extract may have a synergistic effect with echinochrome A to reduce its cytotoxicity. The reason why echinochrome A exhibited cytotoxicity towards the Saos-2 cells is unclear, and further investigation should be carried out to find out the mechanism of the toxic effect of PHNQs on human cells.

The stability of PHNQs in media should be taken into consideration when the cytotoxicity and mineralization nodule formation effect of the PHNQs were tested. Sung et al. [30] did not test the stability of echinochrome A in media under their cell culture conditions, but they tested the cytotoxicity of an ‘exhausted’ form of echinochrome A, prepared by exposing echinochrome A to air and light for 48 h without any ROS-scavengers. It was found that there was no difference in cytotoxicity on either A7r5 cells or H9c2 cells in the presence of up to 50 μM exhausted echinochrome A. In the present study, the stability of PHNQs and echinchrome A was evaluated (Appendix B) and it was found that they were relatively stable in the cell culture conditions when they were in the dark, when either in the presence or absence of CaCl_2_. In the mineralization assays, the effect of echinochrome A and Ca ions, or of PHNQ and Ca ions was evaluated. CaCl_2_ can supply Ca ions for mineralization of bone, and previous studies have clearly indicated that an elevated concentration of calcium is vital for the mineralization process [31]. It would be worth finding out whether there is an effect on the cytotoxicity when adding CaCl_2_ to echinochrome A and PHNQ extracts. In the present study, adding CaCl_2_ (0.5–4.0 mM) to PHNQ did not change the cytotoxicity of PHNQ to Saos-2 cells while it did increase the number of viable cells for some concentration groups, but the reason behind this needs further investigation. It is very important that the PHNQ concentrations tested did not show any cytotoxic effect on the Saos-2 cells. The in vitro cytotoxicity assessment was simpler, faster and less expensive than the human or animal in vivo counterparts. By being non-toxic to Saos-2 cells, it indicated that PHNQ extracts may be non-toxic to osteoblastic cells, which suggests the possibility for further investigation of whether they have the potential for use as therapeutic agents for bone health. However, it should be noted that the results generated by an in vitro model system cannot be considered the same in terms of uptake of nutrients and metabolism compared to what takes place in an animal and human.

The mineralization of extracellular matrix and the formation of mineralized nodules is indicative of the final stages of osteoblast differentiation [16]. Mineralization of the matrix synthesized by a monolayer of Saos-2 cells was analysed with the xylenol orange and the von Kossa staining methods. Calcified tissue formation was clearly observed by eye after 10 days of culture with CaCl_2_ and representative examples of mineralized nodule formation stained by xylenol orange and von Kossa at day 20 is shown in Figure 5. In the study of Chang et al. [31], it was shown that the onset of mineralization starts around 6 to 8 days after the rise in detectable calcium in the cell layer. In the present study, day 10 was used as the first timepoint to measure the effect of mineralized nodule formation. Wang et al. [32] found that suitable nodule identification was possible beyond day 15. Some natural products such as *Puerariae radix* extract have been reported to induce mineralized nodule formation at 14 days incubation of Saos-2 cells [33]. Thus, in the present study, it was decided to use day 10, day 15 and day 20 timepoints at which to analyse the formation of mineralized nodules of different samples, but only day 20 was analysed with the von Kossa staining method because it requires cell termination and fixation.

Despite the disadvantage of the von Kossa staining method that involves the termination of the cells in culture, as commented on in the introduction, it is still considered as the standard method to visualize mineralization in osteoblast cells [30,34]. The high correlation between the xylenol orange fluorescent staining method and the von Kossa method further confirmed the reliability of the fluorescent staining method. This enabled the observation of mineralization in living cell cultures at different time points (day 10, day 15, and day 20). In the following study, the plate reader data were used to quantitatively analyse the mineralization effect. The mineralized nodule formation was further confirmed with images from the von Kossa and fluorescent staining methods.

PHNQs belong to a family of naphthoquinone compounds that each contain several hydroxyl groups. Vitamin K also comprises a family of naphthoquinones and has been used as a therapy to prevent bone mineral loss and reduce risk of fracture in osteoporotic patients [35]. Vitamin K_1_ has been shown to retard bone loss and improve bone health in human trials [35,36,37]. Vitamin K_2_ has been demonstrated to have the effect of osteoporosis prevention and has been clinically utilized. It exerts its protective effects by promoting osteoblast differentiation and mineralization [38]. Menaquinone-7, a derivative of vitamin K_2_ containing seven isoprene units, was found to promote osteoblast bone formation in vitro, and there was a significant increase in alkaline phosphatase activity, DNA content, and calcium content in osteoblast Saos-2 cells [38]. The results indicate that menaquinone-7 has a stimulatory effect on osteoblast Saos-2 cells in vitro. In another study, Yamaguchi et al. (2001) showed that protein content, alkaline phosphatase activity, osteocalcin and DNA content in osteoblast MC3T3-E1 cells after culturing for 24 h in a serum-free medium containing menaquinone-7 at 10^−7^–10^−5^ M were significantly increased. Similar to vitamin K, PHNQ extracts had a tendency to increase mineralized nodule formation compared to that of cells only. The mechanism behind the effect needs further investigation. It is important to note that the decrease in the formation of mineralized nodules was only observed with the echinochrome A group but not the PHNQ extract group, and this may be due to the cytotoxicity of echinochrome A to Saos-2 cells.

Recent studies have indicated an association between ROS-induced oxidative stress and the detrimental effects on bone-forming osteoblasts [39]. Oxidative stress was found to be one of the most important contributors to the pathogenesis of osteoporosis via its role in detrimental effects such as oxidative stress on bone-forming osteoblasts. However, even though PHNQ compounds exhibit antioxidant activity [8], it should be noted that the antioxidant activity of the PHNQ extract cannot solely explain the mechanism of the mineralized nodule formation effect. Echinochrome A had a higher antioxidant activity compared to the PHNQ extract [8], whereas it did not increase the mineralized nodule formation effect on Saos-2 cells at all the testing concentrations. Therefore, further investigation should be carried out to find out the mechanism of mineralized nodule formation effected by the PHNQ extract.

According to the results shown in Figure 7B, PHNQ extract at three different concentrations did not have a significant effect on mineralized nodule formation compared with the cells only control (*p* < 0.05). In addition, treatment with CaCl_2_ increased mineralized nodules in a dose-dependent manner at concentrations in the range of 1.5 to 2.5 mM, as mentioned in a previous section (Figure 5A). Whether adding 1.5–2.5 mM CaCl_2_ to different concentrations of PHNQ affected the mineralized nodule formation in Saos-2 cells was evaluated. It should be noted that although the sea urchin shell and spine is composed of mainly mineral, the PHNQs were extracted with organic solvent, and hence only a trace amount of calcium would have been present in the PHNQ extract. The concentration of CaCl_2_ in media containing the highest concentration of PHNQ extract was determined to be below 0.17 µM, which was far below the amount of the CaCl_2_ added to the PHNQ extract. Yamauchi et al. [40] reported a stimulatory effect of calcium (2.8–3.8 mM) in the mineralization process of MC3T3-E1 cells. The authors found that mineralization of mouse osteoblast MC3T3-E1 cells increased in a dose-dependent manner when the cells were exposed to high calcium (2.8 and 3.8 mM) compared with a control-level treatment (1.8 mM) [40]. A previous study also showed that high calcium induced both a chemotaxis effect and the proliferation of MC3T3-E1 cells [41]. A pilot study undertaken by Chang et al. [31] showed that maximum mineralization occurred when the medium was supplemented with a final concentration of 3.95 mM Ca^2+^ for rat calvarial osteoblast-like cells. These results are similar to those obtained in the present study even though the cell lines used were not the same. Bone formation is initiated by the migration of pre-osteoblasts into resorption pits at the end of osteoclast bone resorption. Large amounts of calcium are released from the mineralized bone matrix during osteoclast resorption, raising the level of calcium in the vicinity of resorption sites [40]. It is possible that the extracellular calcium-sensing receptor, which is expressed in various bone-marrow-derived cell lines and plays an important role in stimulating their proliferation and chemotaxis, could sense the high level of calcium, thereby providing a signal for new bone nodule formation [40]. A significant (*p* < 0.05) increase in mineralization was observed when PHNQs (62.5 µg/ mL) were supplemented with 1.5 mM CaCl_2_. This result indicates that PHNQs have the potential to improve the formation of the bone mineral phase in vitro, and future research in animal models is warranted.

## 4. Materials and Methods

### 4.1. The Source of Sea Urchin Spine Samples and Echinochrome A Standard

Sea urchins (*E. chloroticus*) were collected from around the southern coast of the South Island, New Zealand, by a commercial company (Cando Fishing Ltd., Bluff, New Zealand). The shells with spines were couriered to the University of Otago on the same day of harvesting, and were washed with cold water, air-dried in the dark and then the spines were separated from shell and ground into powder (Jingangdikai JG100 grinder, Jingangdikai Co., Guangdong, China). The powder was sieved (mesh size 450 µm) and stored at −20 °C in the dark. The echinochrome A was kindly provided by Professor Mishchenko from G.B. Elyakov Pacific Institute of Bioorganic Chemistry, Far Eastern Branch of Russian Academy of Sciences, Vladivostok, Russia.

### 4.2. Preparation of Sea Urchin Spine Crude Extract

The dried spine powders were dissolved by gradually adding 6 M HCl to achieve a final solid to liquid ratio of 1:5 (*w*/*v*) in the dark at room temperature according to the method used in a published study [3]. The mixture was centrifuged (13,300× *g*, 20 min, 4 °C), and aliquots of the clarified supernatant containing the PHNQ pigments were extracted three times with the same volume of ethyl acetate. The organic solvent extracts containing the pigments were washed with Milli-Q water to remove any residual acid and then dried over anhydrous sodium sulphate. The organic solvent was evaporated to dryness under reduced pressure in a rotary evaporator at 45 °C in the dark. The PHNQ pigments were then re-dissolved at 10 mg/mL in DMSO and used as a stock solution for cell-based bioactivity assays.

### 4.3. Characterisation of PHNQ Compounds Using HPLC with Diode-Array Detection and Mass Spectrometry (HPLC-DAD/MS)

A full description of the characterisation of the compounds is reported in Hou et al. [3]. The method was conducted according to the method of Vasileva et al. [29]. Before analysis, samples were filtered through a 0.2 μm PTFE syringe filter (Axiva). The injection volume was 2 μL. The HPLC used was a Shimadzu system with a diode-matrix SPD-M20A (Shimadzu USA Manufacturing Inc., Canby, USA) connected to a mass-spectrometry (MS) detector LCMS-2020 (Shimadzu Corp., Kyoto, Japan). The separation was carried out on a Discovery HS C_18_ column (150 × 2.1 mm, 3 μm particle size, Supelco, Bellefonte, PA, USA) with a Supelguard Ascentis C_18_ pre-column (2 × 2.1 mm, 3 μm particle size, Supelco, Bellefonte, PA, USA) using a binary gradient of H_2_O (A): acetonitrile (B) with the addition of 0.1% methanol, at a flow rate of 0.2 mL/min and column temperature of 40 °C. The gradient was as follows: 0–6 min, 10–40% (B); 6–11 min, 40–100 % (B); 11–12 min, 100% (B), 12–13 min, 100–10% (B); and 13–17 min, 10% (B). The chromatograms were recorded at 254 nm. Mass spectra were acquired in the electrospray ionization (ESI) mode at atmospheric pressure, recording negative ions (1.50 kV) in the m/z range of 100–800.

### 4.4. Determination of Cytotoxicity of PHNQ by MTT Assay Using Saos-2 Cells (48 h Incubation Time)

The cytotoxicity of the PHNQ extract was determined using the MTT assay, as described [42]. Human sarcoma osteogenic (Saos-2 cells) were sourced from the American Type Culture Collection (ATCC^®®^) (Manassas, VA, USA), ATCC^®®^ number HTB-85™. Saos-2 cells were thawed from liquid nitrogen storage (passage number: 12) and then cultured in growth media at 37 °C in an atmosphere of 95% air and 5% CO_2_ in growth media (Minimum Essential Medium(MEM)-α supplemented with 10% (*v*/*v*) heat inactivated FBS and 1% (*v*/*v*) antibiotic-antimycotic solution). The Saos-2 cells were seeded in 96-well tissue culture plates at a density of 3 × 10^5^ cells/mL (100 µL each well) in growth media (growth media supplemented with 10 μM dexamethasone, 50 μg/mL L-ascorbic acid 2-phosphate sesquimagnesium salt hydrate, and 10 mM β-glycerolphosphate pentahydrate disodium salt). Once the cells reached 80-100% confluence (the cell confluence was observed visually using an inverted light microscope), the media were changed to growth media with or without PHNQ at different concentrations (1000, 500, 250, 125, 62.5, and 37.25 µg/mL). The cells were maintained in the growth media at 37 °C in an atmosphere of 95% air and 5% CO_2_ for 48 h. SDS was used as a positive control. Following incubation, media with or without PHNQ extract were removed, and the cells were washed twice gently in pre-warmed (37 °C) PBS. MTT (10 µL) (Molecular Probes, Life Technologies M6494; Thermo Fisher Scientific Inc., Auckland, New Zealand) at 5 mg/mL and 50 µL of growth media were added to each well, gently mixed, and the plate was re-incubated at 37 °C with 95% air and 5% CO_2_ for another 4 h. The solution in each well was then removed and 100 µL of DMSO was added. After a 10 min incubation, the absorbance of the solution in each well of the plate was determined on a Varioskan™ Flash Multimode Reader (Thermo Fisher Scientific Inc., Auckland, New Zealand) at 570 nm. Results were reported as absorbance measured at 570 nm. The viable cell percentage (%) was calculated in the following equation. Data were from three independent replicates, each concentration tested in duplicate

$$\mathrm{Viable}\;\mathrm{cell}\;\mathrm{percentage}\;\left(\%\right)=\frac{A_{\mathrm{sample}}-A_{\mathrm{DMSO}}}{A_{\mathrm{control}}\;}\times 100\%.$$

where A_sample_ is the absorbance data of Saos-2 cell viability measured by the MTT assay treated by PHNQ extract, A_control_ is the absorbance data of Saos-2 cells alone, A_DMSO_ is the absorbance data of DMSO control.

### 4.5. Determination of Cytotoxicity of PHNQ, Echinochrome A, CaCl_2_, and PHNQ or Echinochrome A Supplemented with CaCl_2_ by MTT Assay Using Saos-2 Cells (21 days Incubation Time)

The cytotoxicity of the PHNQ extract, echinochrome A, CaCl_2_, and PHNQ or echinochrome A supplemented with CaCl_2_ was determined using the MTT assay, as described in Section 4.4. Once the cells reached 80 to 100% confluence, they were passaged for seeding in 96-well tissue culture plates (passage number: 13; cells that were used were not passaged more than 20 times) for MTT and mineralization assays. Saos-2 cells were seeded in 96-well tissue culture plates at a density of 1 × 10^4^ cells/mL (100 µL per well) and cultured in growth media in a humidified atmosphere at 37 °C with 5% CO_2_. Once the cells were at 80 to 100% confluence (2–3 days), the day that the plated cells became confluent was designated as day 0 and the media were changed to treatment media (growth media supplemented with 10 μM dexamethasone, 50 μg/mL l-ascorbic acid 2-phosphate sesquimagnesium salt hydrate, and 10 mM β-glycerolphosphate pentahydrate disodium salt, 100 µL per well), with or without different concentrations of added test chemicals (details shown in Table 1), and the cells were grown for 21 days with the media changed every 2–3 days. The liquid in each well was then removed and the cells were washed twice gently in pre-warmed (37 °C) PBS. Subsequently, MTT (10 µL) at 5 mg/mL and 50 µL of growth media were added to each well, gently mixed, and the plate was re-incubated at 37 °C with 95% air and 5% CO_2_ for another 4 h. The solution in each well was then removed and 100 µL of DMSO was added. After a 10 min incubation the absorbance of the solution in each well of the plate was determined on a Varioskan™ Flash Multimode Reader at 570 nm.

### 4.6. Determination of Mineralized Nodule Formation by Xylenol Orange Staining

The effect of PHNQ extract on mineralized nodule formation was determined using the xylenol orange staining method [32,43]. The cells were seeded on 96-well tissue culture plates at a density of 1 × 10^4^ cells/mL (100 µL) in growth media. Once cells were at 80–100% confluence (observed visually using an inverted light microscope (CK40, Olympus, Tokyo, Japan)), the growth media were then changed to treatment media (100 µL for each well) with or without different concentrations of CaCl_2_, PHNQ extract, and echinochrome A, or PHNQ extract with CaCl_2_, or echinochrome A with CaCl_2_ (the final concentrations are shown in Table 1) and the cells were grown for 9 days with media change every 2–3 days. On day 10, cells were stained with 0.2 µL of 10 mM xylenol orange in each well (20 μM in the final media) and incubated for a further 24 h. After incubation, the cells were washed three times with 50 µL pre-warmed (37 °C) PBS for each well before reading by a VarioskanFlash plate reader. Pre-warmed (37 °C) PBS, rather than treatment media, was added to each well for reading by a VarioskanFlash plate reader with excitation and emission wavelengths of 440 and 610 nm, respectively, in order to eliminate the influence of PHNQ in the treatment media. After reading by a VarioskanFlash plate reader, prior to microscopic examination and photography, the PBS was removed, and cultures received fresh medium without xylenol orange fluorochrome to avoid production of a non-specific fluorescence background. Then, images were recorded for further analysis with a Nikon DS-Qi2 Camera fitted with a Nikon (ECLIPSE, Ti2) (Coherent Scientific Pty. Ltd., Hilton SA 5033, Australia) inverted fluorescence (or light) microscope. After that, the cells were incubated until day 14 with a media change on day 12. Then the cells were stained on day 14 using the same method followed by further washing, reading by a VarioskanFlash plate reader and photographed with a Nikon DS-Qi2 Camera fitted with a Nikon (ECLIPSE, Ti2) inverted fluorescence (or light) microscope on day 15. Another measurement was carried out on day 20 after staining on day 19 and washing on day 20. The results were reported as percentage of fluorometric reading of treatment group compared to the cells-only control. Data were from three independent replicates, and each concentration tested in duplicate

$$\mathrm{Percentage}\;\mathrm{of}\;\mathrm{cells}\;\mathrm{only}\;\mathrm{control}\;\left(\%\right)=\frac{{\;F}_{\mathrm{sample}}-F_{\mathrm{vehicle}}\;}{F_{\mathrm{control}}}\times 100\%.$$

where F_sample_ is the fluorescence of Saos-2 cells exposed to CaCl_2,_ PHNQ extract, and echinochrome A, or PHNQ extract with CaCl_2_, or echinochrome A with CaCl_2_, F_control_ is the fluorescence of Saos-2 cells alone, F_vehicle_ is the fluorescence of DMSO control.

### 4.7. Determination of Mineralized Nodule Formation by von Kossa Staining

The presence of mineralized nodules observed by xylenol orange staining was further evaluated using the von Kossa method [32]. For the von Kossa silver nitrate staining method, the media were removed, and the cells were rinsed twice with deionised water prior to being fixed with cold methanol for 15–20 min. After fixation, the cells were rinsed again with deionised water. An aliquot (50 µL) of 5% (*w*/*v*) silver nitrate was then added and the cells were incubated with the silver nitrate for 20 min at room temperature. The staining solution was then removed, and the cells were rinsed again with deionised water. The stained plates were then inspected visually for nodule formation using an inverted light microscope (Nikon, ECLIPSE, Ti2). Photographs of the plates were taken using a Nikon DS-Qi2 Camera fitted to the inverted light microscope.

### 4.8. Statistical Analysis

The data were analysed using analysis of variance (ANOVA) using Minitab 17.0 Software (Minitab Pty Ltd., Sydney, Australia). One-way ANOVA were used to assess the effect of PHNQ, echinochrome A and CaCl_2_ concentration at a given time on the cytotoxicity to Saos-2 cells. The general linear model protocol was used to determine the effects of the PHNQ, echinochrome A and CaCl_2_ concentrations and different timepoints on the mineralization effect on Saos-2 cells. The Crossed Gage R&R Study (method of analysis: ANOVA) was used to analyse the repeatability of the data in Section 2.4. The EC_50_ for the PHNQ and for echinochrome A were determined by fitting the obtained data using polynomial curve in GraphPad Prism 8.4.3 (GraphPad Software, San Diego, CA, USA). The results are reported as mean ± standard error of the mean and significant differences among the means were determined using Tukey’s honesty test at *p*-value < 0.05. Data were obtained from at least three independent experiments.

## 5. Conclusions

In conclusion, the present study suggests that PHNQ extract is non-toxic to osteoblast cells at concentrations below 62.6 µg/mL, over 21 days incubation with the cells. A high concentration of PHNQ extract (62.6 µg/mL) plus 1.5 mM CaCl_2_ synergistically increased mineralized nodule formation that does not happen in the presence of high concentrations of PHNQ extract if administered separately. This indicates that PHNQ extract has the potential to improve the formation of the bone mineral phase sand could potentially be used as a therapeutic agent for the prevention or treatment of osteoporosis. It is worth mentioning the limitations of using the Saos-2 cell line model for evaluation of mineralization effect. Even through the Saos-2 cell line showed similar mineral potential and gene regulation with primary human osteoblast (HOb) cells, it demonstrated a higher proliferation rate. In addition, the results generated by cell culture (in vitro model system) cannot be considered the same in terms of uptake of nutrients and metabolism compared to what takes place in an animal and human [44]. In the future, further sub-cellular characterization needs to be done to confirm the mineralization effect of PHNQ in Saos-2 cells. In addition, the data from the in vitro cell line system should be considered only indicative, and further animal trials should be conducted in the future.

## Figures and Tables

**Table 1:** Sample details for MTT and mineralization assays.

Sample Name	Final Concentration in Media
CaCl_2_ control	CaCl_2_ 0–4.0 mM
High concentration of echinochrome A	62.5 µg/mL
Medium concentration of echinochrome A	31.25 µg/mL
Low concentration of echinochrome A	15.625 µg/mL
High concentration of PHNQ	62.5 µg/mL
Medium concentration of PHNQ	31.25 µg/mL
Low concentration of PHNQ	15.625 µg/mL

**Figure A1:**
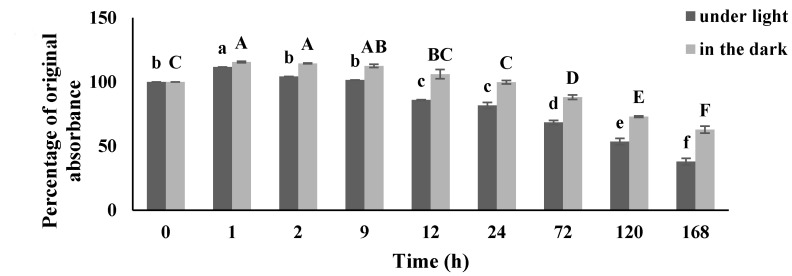
The stability of PHNQ extract in cell culture media. The percentage of the original absorbance at 340 nm of PHNQ extract (in cell culture media at the concentration of 62.5 µg/mL) at different time points in media at room temperature either in the dark or under the light. Means with different letters for the group under light (a–f) and in the dark (A–F) are significantly different (*p* < 0.05).

## Appendix A

Images taken from mineralized nodule formation assays were analysed by ImageJ (ImageJ bundled with 64-bit Java 1.8.0_112) in a quantitative manner. For the xylenol orange fluorescent staining, the image type was set as 8-bit and then the image was inverted before calibration and setting the threshold. Then a rectangle was selected from the same position of all of the images and the percentage of area was measured. For the von Kossa staining method, RGB stack was run before setting up the threshold and then the image was converted to mask. The same size of rectangle was selected from all of the images from the same position and the percentage of area (fluorescent staining area/selected rectangle area) was measured. The results were reported as percentage of area of treatment group in the image compared to the cells-only control.

Results were reported as percentage of the cells only control.

$$\mathrm{Percentage}\;\mathrm{of}\;\mathrm{cells}\;\mathrm{only}\;\mathrm{control}\;\left(\%\right)=\frac{\mathrm{Area}\;\mathrm{of}\;\mathrm{sample}}{\mathrm{Area}\;\mathrm{of}\;\mathrm{control}}\times 100\%.$$

## Appendix B

### Appendix B.1. PHNQ Stability in Cell Culture Media

The determination of PHNQ stability in cell culture media was carried out according to the method described in a previous study [3]. PHNQ extracts were dissolved in DMSO at a concentration of 10 mg/mL. Then the PHNQ stock solution was diluted in treatment media to a final concentration of 62.5 µg/mL. Aliquots of PHNQ diluted solution were stored at room temperature (20 ± 2 °C) either in the dark or under natural light. The absorbance of the PHNQ solutions at 340 nm was measured at 0, 1, 2, 9, 12, 24, 72 h. Then a 96-well plate was prepared with 100 µL of treatment media with different concentration of PHNQ extract and CaCl_2_ (the details shown in Table 1) and stored in an atmosphere of 95% air and 5% CO_2_ at 37 °C for 3 days in the dark (equivalent to the cell culture conditions). The absorbance of the PHNQ solutions at 340 nm was measured both before and after the incubation. The results are reported as percentage of the initial absorbance.

$$\mathrm{Percentage}\;\mathrm{of}\;\mathrm{the}\;\mathrm{original}\;\mathrm{absorbance}\;\mathrm{at}\;340\;\mathrm{nm}\;\left(\%\right)=\frac{A_{\mathrm{PHNQ}}-A_{\mathrm{media}}\;}{A_{\mathrm{PHNQ}\;0}-A_{\mathrm{media}\;0}}\times 100\%$$

where A_PHNQ_ is the absorbance of the media with PHNQ after three days incubation; A_media_ is the absorbance of the media only, after three days incubation; A_PHNQ 0_ is the absorbance of the media with PHNQ, before incubation (original absorbance); A_media 0_ is the absorbance of the media without PHNQ before incubation (original absorbance).

### Appendix B.2. Results of PHNQ Stability in Cell Culture Media

The stability of PHNQ extract at 62.5 µg/mL in cell culture media was first evaluated at room temperature as a preliminary experiment. The absorbance of cell culture media containing PHNQ extract under natural light was decreased by about one third compared to the initial absorbance (Figure A1), while the absorbance of the PHNQ in the dark decreased about 10% after 72 h. Compared with the earlier stability study reported by Hou et al. [3], PHNQs were found to be more stable in cell culture media than in methanol solution. It should be noted that the different concentration of PHNQ extract in the two testing systems (62.5 µg/mL PHNQ extract in media, compared to 1 mg/mL in methanol) might have contributed to the difference in stability.

As discussed in a previous study [3], the impact of natural light on the stability of PHNQ has been considered as one of the parameters that can cause degradation of PHNQ, even though it was in a cell culture media system. Thus, the PHNQ sample preparation should be carried out to avoid light as much as possible. The majority of PHNQ (88%) was still stable in the cell culture media after 72 h in the dark.

**Table A1:** The stability of PHNQ extract in cell culture media under cell culture conditions in the presence of CaCl_2_ *.

CaCl_2_ (mM)/PHNQ Concentration (µg/mL)	15.625 (Low)	31.25 (Medium)	62.5 (High)
Ca 0 mM	73.86 ± 14.98 ^a^	67.21 ± 10.98 ^b^	84.38 ± 4.93 ^b^
Ca 0.5 mM	77.93 ± 17.65 ^a^	80.90 ± 10.71 ^ab^	71.46 ± 3.26 ^ab^
Ca 1.0 mM	69.99 ± 8.80 ^a^	73.67 ± 3.71 ^ab^	74.91 ± 3.46 ^b^
Ca 1.5 mM	68.33 ± 19.98 ^a^	68.66 ± 8.92 ^b^	57.13 ± 5.27 ^b^
Ca 2.0 mM	66.30 ± 22.70 ^a^	68.00 ± 7.99 ^b^	80.85 ± 7.68 ^b^
Ca 2.5 mM	66.19 ± 16.93 ^a^	70.00 ± 10.38 ^ab^	63.87 ± 8.10 ^b^
Ca 3.0 mM	65.41 ± 16.98 ^a^	68.26 ± 13.20 ^b^	86.25 ± 13.73 ^b^
vehicle control **	103.2 ± 5.05 ^a^	106.77 ± 3.19 ^a^	106.24 ± 8.03 ^a^

* Percentage of original absorbance (%) was calculated based on the equation in Appendix B.1. PHNQ extract (in cell culture media) at high, medium or low concentrations, with different amounts of CaCl_2_, at 37 °C in an atmosphere of 95% air and 5% CO_2_ in the dark after 72 h incubation. Means with different letters within the same column are significantly different (*p* < 0.05). ** Same volume of DMSO was used as treatment group for the treatment in vehicle control group.

Considering that PHNQ extracts were incubated at 37 °C, under an atmosphere of 95% air and 5% CO_2_ with Saos-2 cells in the presence of CaCl_2_, the stability of PHNQ extracts was examined under cell culture conditions in the presence of CaCl_2_. Because the media were changed every two to three days, the incubation time was set as 72 h. After 72 h incubation, the absorbance of the PHNQ extract decreased by about one third in both the high and medium PHNQ concentrations (Table A1) in media. With the lowest PHNQ concentration (15.625 µm/mL) in media, there was more variation but at least 60% of the original absorbance was retained after incubation (Table A1). The amount of CaCl_2_ present in the culture media did not have any effect on the stability of PHNQ extract in the cell culture media.

It is important to note that the stability of echinochrome A and PHNQ extract in the presence of calcium should be taken into consideration in the following experiments. According to a previous study [45], at alkaline pH, the addition of calcium ions to echinochrome A aqueous solution led to an increase in the auto-oxidation rate. The authors suggested that complexing of Ca^2+^ leads to a decrease in the dissociation constant of PHNQs [45]. In the presence of Ca^2+^, PHNQs are present in polyvalent anion forms in the aqueous solution. In the polyvalent anion form, PHNQs are sensitive to oxygen and are more likely to be auto-oxidised [45]. Lebedev et al. [46] also suggested that the addition of Ca^2+^ to PHNQs (echinochrome A) led to the formation of stable calcium-semiquinone adducts, that would potentially shift the oxidation-reduction equilibrium to the oxidation side. It should be noted that in the study of Lebedev et al. [45], when the pH was under 7.5, the rate of echinochrome A oxidation was still very low compared to that occurring at higher pH. The stability of PHNQ extract with different concentrations of CaCl_2_ showed that the majority of the PHNQs remained in solution in the media under the cell culture conditions and therefore should be available to effect biological activity.

The UV spectra generated from the pigment extracts had maxima at 270, 340, and 475 nm [1]. The wavelength of 340 nm was chosen in this study rather than 475 nm to avoid absorbance contribution of the media, as the media was pink coloured which has absorbance at around 475 nm. Although HPLC analysis is more specific than UV spectra for the analysis of PHNQ compounds due to the fractionation achieved, the recovery of PHNQs from media was not effective using organic solvent extraction. Overall, the majority of the PHNQ remained in the media under the cell culture conditions and would be still available to exert biochemical effects.
